# Correlation of Retinal Nerve Fibre Layer Thickness and Spontaneous Retinal Venous Pulsations in Glaucoma and Normal Controls

**DOI:** 10.1371/journal.pone.0128433

**Published:** 2015-06-04

**Authors:** S. Mojtaba Golzan, William H. Morgan, Dana Georgevsky, Stuart L. Graham

**Affiliations:** 1 Department of Clinical Medicine, Faculty of Medicine and Health Sciences, Macquarie University, Sydney, Australia; 2 Lions Eye Institute, University of Western Australia, Perth, Australia; Duke University, UNITED STATES

## Abstract

**Purpose:**

To study the relationship between amplitude of spontaneous retinal venous pulsatility (SRVP) and retinal nerve fibre layer (RNFL) thickness in glaucomatous eyes, and to determine if this parameter may be a potential marker for glaucoma severity.

**Method:**

85 subjects including 50 glaucoma (21 males, 67±10 yrs) and 35 normals (16 males, 62±11 yrs) were studied. SRVP amplitude was measured using the Dynamic Vessel Analyser (DVA, Imedos, Germany) at four regions of the retina simultaneously within one disc diameter from the optic disc—temporal-superior (TS), nasal-superior (NS), temporal-inferior (TI) and nasal-inferior (NI)). This was followed by RNFL thickness measurement using spectral domain optical coherence tomography (Spectralis OCT). The correlation between SRVP amplitude and corresponding sectoral RNFL thickness was assessed by means of non-linear regression (i.e. logarithmic). Linear regression was also applied and slopes were compared using analysis of covariance (ANCOVA).

**Results:**

Greater SRVP amplitude was associated with thicker RNFL. Global SRVP amplitude was significantly lower in glaucoma eyes compared with normals (p<0.0001). The correlation coefficient of the linear regression between RNFL and SRVP at TS, NS, TI and NI quadrants in the glaucoma group were r = 0.5, 0.5, 0.48, 0.62. Mean SRVP amplitude and RNFL thickness for TS, NS, TI and NI quadrants were 4.3±1.5, 3.5±1.3, 4.7±1.6, 3.1±1 μm and 96±30, 75±22, 89±35 and 88±30 μm, respectively. The ANCOVA test showed that the slope of linear regression between the four quadrants was not significant *(p>0*.*05)*. Since the slopes are not significantly different, it is possible to calculate one slope for all the data. The pooled slope equals 10.8 (i.e. RNFL = 10.8SRVP+41).

**Conclusion:**

While SRVP was present and measurable in all individuals, the amplitude of SRVP is reduced in glaucoma with increasing RNFL loss. Our findings suggest the degree of SRVP may be an additional marker for glaucoma severity. Further studies are needed to determine the mechanism of reduction in SRVP, and whether changes can predict increased risk of progression.

## Introduction

Spontaneous retinal venous pulsations (SRVP) result from an interaction between intraocular pressure (IOP), retinal venous pressure (RVP) and cerebrospinal fluid pressure (CSFp). With elevated CSFp, raised RVP or reduced IOP, the intravascular pressure gradient across the prelaminar and retrolaminar portions of the central retinal vein decreases, leading to cessation of SRVP[1–3]. Morgan et al has demonstrated the dependence of retinal vein pressures upon IOP and CSF pressure [4,5]. He also measured the trans-lamina pressure gradient identifying its strong relationship with IOP and CSF pressure[6] which are both associated with glaucoma[7]. We have shown that IOP and CSFp dynamically contribute towards the amplitude of these pulsations [8]. While a reduction of IOP leads to decreased SRVP, it has also been reported that reduced SRVP is a risk factor for glaucoma[5], and we found an increased risk of progression of glaucoma with absence of visible pulsation [9]. Visible SRVP has been reported in 54% of glaucoma patients compared with 75% and 98% in glaucoma suspects and normals, respectively[5]. Retinal vein pulsation pressure, the threshold IOP at which vein pulsation is visible, is increased in more advanced stages of glaucoma. This suggests an alteration in pulsation properties is occurring during the development of glaucoma. One limitation of these threshold, ophthalmodynamometric measures, is that they can only be performed in approximately 50% of glaucoma subjects. Additionally, glaucoma is known to be strongly associated with optic nerve haemorrhage[10] and central retinal vein occlusion[11] suggesting a potential vascular relationship. Clearly this relationship is a complex one, and as yet the factors defining the generation of SRVPs have not been determined, nor has their relevance to glaucoma pathogenesis.

Different studies have investigated the nature of SRVPs[12,13]. It was proposed that the trans-laminar pressure gradient (difference between IOP and CSFp) was the main reason for the phenomena[14,15]. Berdahl et al[7] showed that the mean CSFp was significantly higher in the non-glaucomatous eyes (13.0 ± 4.2 mmHg) compared with glaucomatous eyes (9.2 ± 2.9 mmHg, p<0.001). This was later confirmed by Ren et al[14] in a study that found the trans-laminar pressure gradient was significantly higher in glaucoma patients (12.5+/-4.1 mmHg) compared with controls (1.4+/-1.7 mmHg, p<0.001). This study also demonstrated that visual field (VF) loss negatively correlated with the height of the CSFp and positively correlated with the trans-laminar pressure gradient.

The purpose of this study, was to investigate the relationship between amplitude of SRVP and RNFL thickness at four regions (i.e. temporal-superior (TS), nasal-superior (NS), temporal-inferior (TI) and nasal-inferior (NI)) of the retina in glaucomatous and normal eyes. We used RNFL thickness to stage severity of glaucoma in those sectors and amplitude of pulsations to give a quantitative measure of pulsation in all subjects.

## Methodology

### Data Collection

50 open angle glaucoma patients (21 male, 67±10 yrs) and 35 healthy volunteers (16 males, 62±11 yrs) were included in the study. All subjects went under a series of ophthalmic tests including slit lamp examination, IOP measurement (Goldman tonometry), measurement of SRVP amplitude using the Dynamic Vessel Analyzer (DVA, Imedos, Germany) and RNFL thickness measurement using Spectralis Optical Coherence Tomography (OCT, Heidelberg, Germany). A 75D lens was used to observe the presence or absence of SRVP prior to DVA measurements.

Glaucoma subjects were identified as having definite glaucomatous changes in the neuro-retinal rim, 3 patients did not yet have visual field defects, while the remaining 47 subjects had corresponding visual field changes with MD range 0–16 dB. A past history of acute angle closure, retinal vein occlusion or other retinal pathology was an exclusion. Normals were included if they had no history of eye disease, a normal fundus on ophthalmoscopy with no visible vascular changes. All subjects had no symptoms or signs of raised CSFp, and no history of diabetes.

### Experimental Paradigm

**Fig 1:**
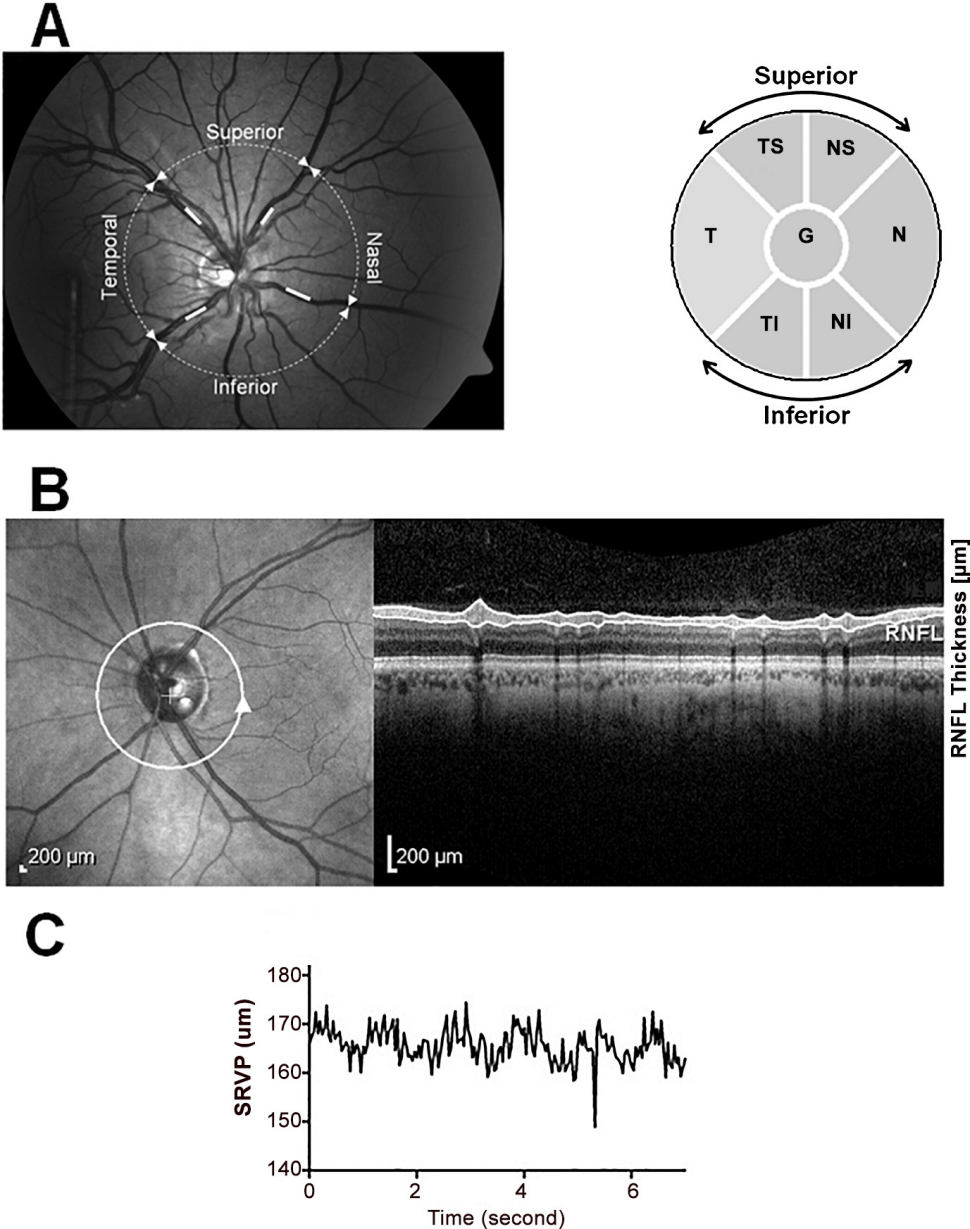
Sites of SRVP amplitude and RNFL thickness measurement. A) Left- SRVP amplitude measurement at TS, NS, TI and NI sectors and Right- six sectors used for RNFL thickness measurements on OCT. B)Left-Site of RNFL measurement, Right- Cross section of retinal layers under the white circle including RNFL. C) Raw trace of SRVP.

Pupils of all subjects were dilated 20 minutes prior to the tests using Tropicamide 1% (Alcon, Texas, USA). SRVP was measured at four sites within 1 disc diameter from optic disc (i.e. temporal-superior (TS), nasal-superior (NS), temporal-inferior (TI) and nasal-inferior (NI)) for 100 sec at a 25 Hz sampling frequency (Fig 1). From the 100 sec recorded data a 20 sec window with the highest signal to noise ratio was selected for further processing. All obtained SRVP recordings were then passed through a low pass filter with a cut off frequency of 3 Hz. Once filtered, peak and troughs of the signal was detected and the difference in these two values was designated as the amplitude of SRVP.

OCT scans were taken at 30° field of view centred on the optic disc. The Spectralis built-in software was used to segment the OCT B-scans and measure RNFL thickness at the relevant four sites (Fig 1).

To evaluate reproducibility and potential fluctuation, 20 of 50 glaucoma patients were selected randomly and re-tested 6 months after their initial visit. This study was performed in accordance with the guidelines of the Tenants of Helsinki and approved by the Macquarie Universities’ ethics committee. Written consent was obtained from the subjects after explanation of the nature and possible consequences of the study.

### Data Analysis

The average SRVP amplitude and RNFL thickness between glaucoma and normal controls were compared using student’s t-test. Linear regression was then used to assess the relationship between SRVP amplitude and RNFL thickness at four quadrants of the optic disc. The RNFL distribution has been reported in many studies and structure /function maps developed [16,17]. Since the sector of the RNFL as defined by OCT does not directly match the distribution of venous drainage for the same location we had to compromise on which sectors to include for correlations. We correlated the SRVP amplitude for the vessel in each of the fours sectors (TS, NS, NI, and TI) with the RNFL thickness within the same sector.

We also performed correlations between the mean of the two superior and two inferior SRVP amplitudes with RNFL mean thickness using the two superior sectors (TS and NS) and the two inferior sectors (TI and NI) (see Fig 1). Further sectoral comparisons were also performed, for example all temporal RNFL sectors with the two temporal vessels, and all nasal sectors with the two nasal vessels. For the pooled grouped data (normal plus glaucoma) we applied an additional logarithmic regression to study the relationship between RNFL thickness and SRVP amplitude for superior and inferior regions. The additional logarithmic regression was performed based on the fact that the average RNFL thickness in normals plateaus at around 110 μm, and we therefore expected a non-linear fit might be more appropriate to evaluate the data.

Analysis of covariance (ANCOVA) was used to compare the slope of the linear regressions. One way Analysis of Variance (ANOVA) was used to measure the difference in the linear regression slope between SRVP amplitude –RNFL thickness across the four regions in glaucomatous eyes. Statistical analyses were performed using Graphpad Prism 6 (La Jolla, CA, USA).

## Results

### SRVP amplitude and RNFL thickness in glaucoma and normal controls

**Fig 2:**
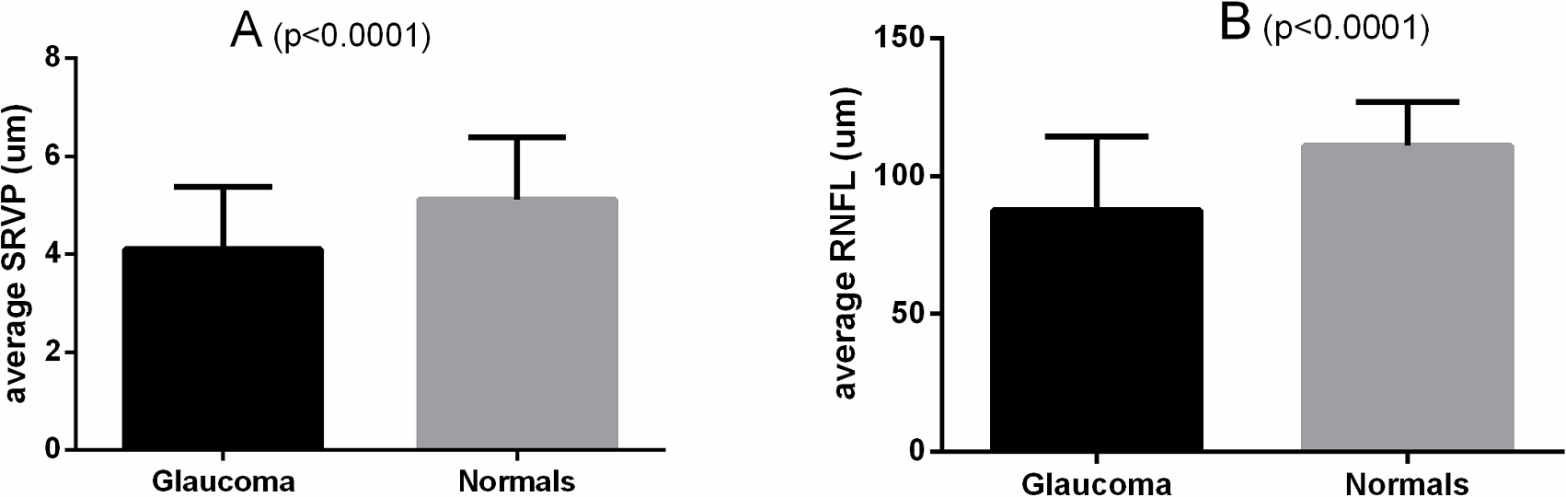
Global (average of all sectors) SRVP amplitude (A) and RNFL thickness (B) in glaucoma subjects vs normal controls (p<0.0001).

Data for all 85 subjects was analysed to determine topographical correlation by individual sectors between SRVP and local RNFL thickness. The global SRVP amplitude (average of all sectors) was significantly lower in the glaucoma group compared with the normal controls (4±1.1 μm vs 5±1.2 μm, p<0.0001). Similarly, global RNFL thickness was significantly lower in the glaucoma group compared with the normal controls (87±26 μm vs 111±15 μm, p<0.0001) (Fig 2).

### Sectoral analysis

When individual sectors of the nerve fibre layer thickness around the optic nerve head were analysed for correlations, we found a positive correlation between SRVP amplitude with RNFL thickness in all four sectors. Fig 1 shows a schematic of the site of the SRVP and RNFL measurements, while Table 1 shows mean SRVP and RNFL measurement at four quadrants of the retina for both normal and glaucoma subjects. Statistical significance between SRVP amplitudes and corresponding RNFL thickness was seen across all retinal sectors. Table 2 shows baseline and follow up data for the glaucoma subjects, which shows a consistent correlation between SRVP and RNFL thickness. Therefore, a consistent regional correlation was observed for each sector.

**Table 1:** Average SRVP and RNFL measurements at 4 sectors of the retina in all glaucoma and normal subjects (correlation coefficient (r) and its p-value for each sector within each group).

	Glaucoma				Normal			
	Temporal- Superior	Nasal- Superior	Temporal- Inferior	Nasal- Inferior	Temporal- Superior	Nasal- Superior	Temporal- Inferior	Nasal- Inferior
SRVP (μm)	4.3±1.5	3.5±1.3	4.7±1.6	3.1±1	5.2±1.4	4.5±1.2	5.4±1.8	4.9±1.3
RNFL (μm)	96±30	75±22	89±35	88±30	119±14	91±16	127±24	102±18
*r*	0.5	0.5	0.48	0.62	0.43	0.53	0.44	0.55
*p value*	<0.0001	<0.001	<0.0001	<0.0001	<0.05	<0.001	<0.05	<0.001

**Table 2:** Average SRVP amplitude and RNFL thickness for baseline and 6 month follow up recordings on 20 glaucomatous eyes (r is correlation coefficient).

	Baseline		6 month follow up	
	Superior	Inferior	Superior	Inferior
SRVP (μm)	3.6±1.3	3.7±1.1	3.1±1.1	3.5±1.5
RNFL (μm)	80±27	76±28	73±27	72±27
*r*	0.66	0.72	0.7	0.71

Linear regression was applied to determine the association between SRVP amplitude and RNFL thickness at four sectors of the retina in glaucoma and normals. The last 2 rows of Table 1 shows the correlation coefficient between SRVP amplitude and RNFL thickness of this regression and its p-value. The best correlations were observed in the NI sector in the glaucoma group (r = 0.62), and occurring in the NI sector also in the normal (r = 0.55).

**Fig 3:**
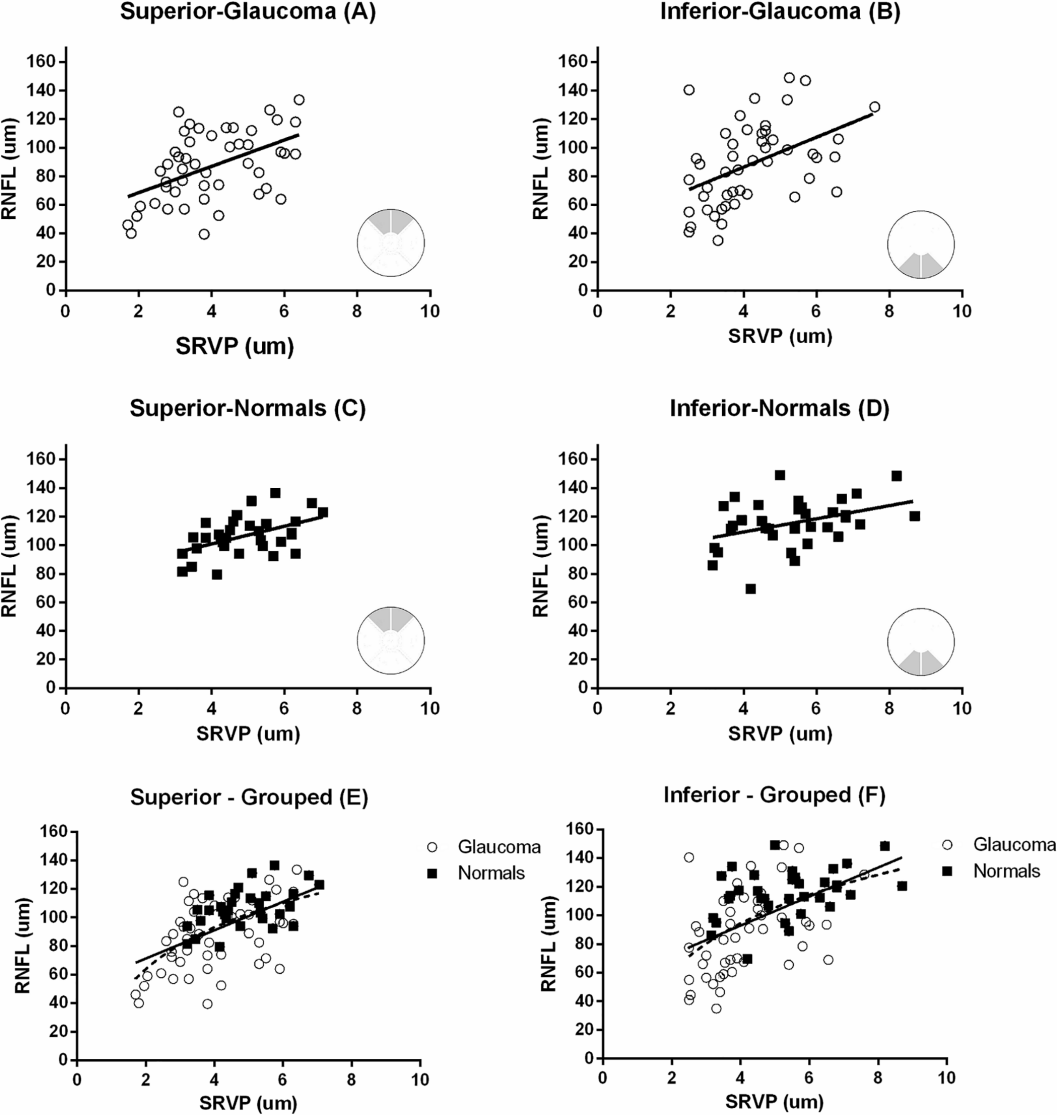
The relationship between mean RNFL thickness and mean SRVP amplitude for corresponding regions (A-D). Grouped analysis for glaucoma subjects and normal controls in both sectors are also shown (E,F). inear regression and correlation coefficient in each sector were A) RNFL = 9.2SRVP+50, r = 0.48, p<0.001, B) RNFL = 10.4SRVP+44.5, r = 0.44, p<0.001, C) RNFL = 6.2SRVP+76, r = 0.48, p<0.001, D) RNFL = 4.6 SRVP+90.8, r = 0.38, p<0.01, E) RNFL = 9.9SRVP+51,r = 0.55, p<0.0001 and F) RNFL = 10.1SRVP+52, r = 0.51, p<0.0001. or the grouped data both a linear and a non-linear regression are shown – solid line = linear, dashed line = non-linear. Goodness of fit for non-linear regression on panels E and F were r^2^ = 0.35 and 0.3 respectively.

Fig 3 shows the positive correlation between average RNFL thickness and average SRVP amplitude at the superior and inferior sector for glaucoma and normal subjects, for both groups individually and when grouped together. Linear regression and correlation coefficient in each sector were; superior-glaucoma) RNFL = 9.2SRVP+50, r = 0.48, p<0.001, inferior-glaucoma) RNFL = 10.4SRVP+44.5, r = 0.44, p<0.001, superior-normals) RNFL = 6.2SRVP+76, r = 0.48, p<0.001, inferior-normals) RNFL = 4.6 SRVP+90.8, r = 0.38, p<0.01, superior-grouped) RNFL = 9.9SRVP+51,r = 0.55, p<0.0001 and inferior-grouped) RNFL = 10.1SRVP+52, r = 0.51, p<0.0001. he pooled data was also analysed with a non-linear logarithmic regression and this gave an r^2^ (goodness of fit) of 0.35for superior and 0.3 for inferior sectors. Fig 3E and 3F show both linear and non-linear regressions.

### Temporal and nasal SRVP

To verify whether the correlation holds for average RNFL thickness on the temporal ornasal sector with the superior and inferior SRVP amplitude on the same side of the optic disc, we correlated mean RNFL for all the temporal sectors with mean SRVP for the two temporal veins (superior and inferior). Results showed that the correlation was no stronger than for individual sectors at r = 0.48. Similarly the correlating nasal three sectors of RNFL including both superior and inferior nasal vessels yielded r = 0.5. he reason for analysing this separately was that there is no absolute demarcation between vascular beds and corresponding RNFL distribution.

**Fig 4:**
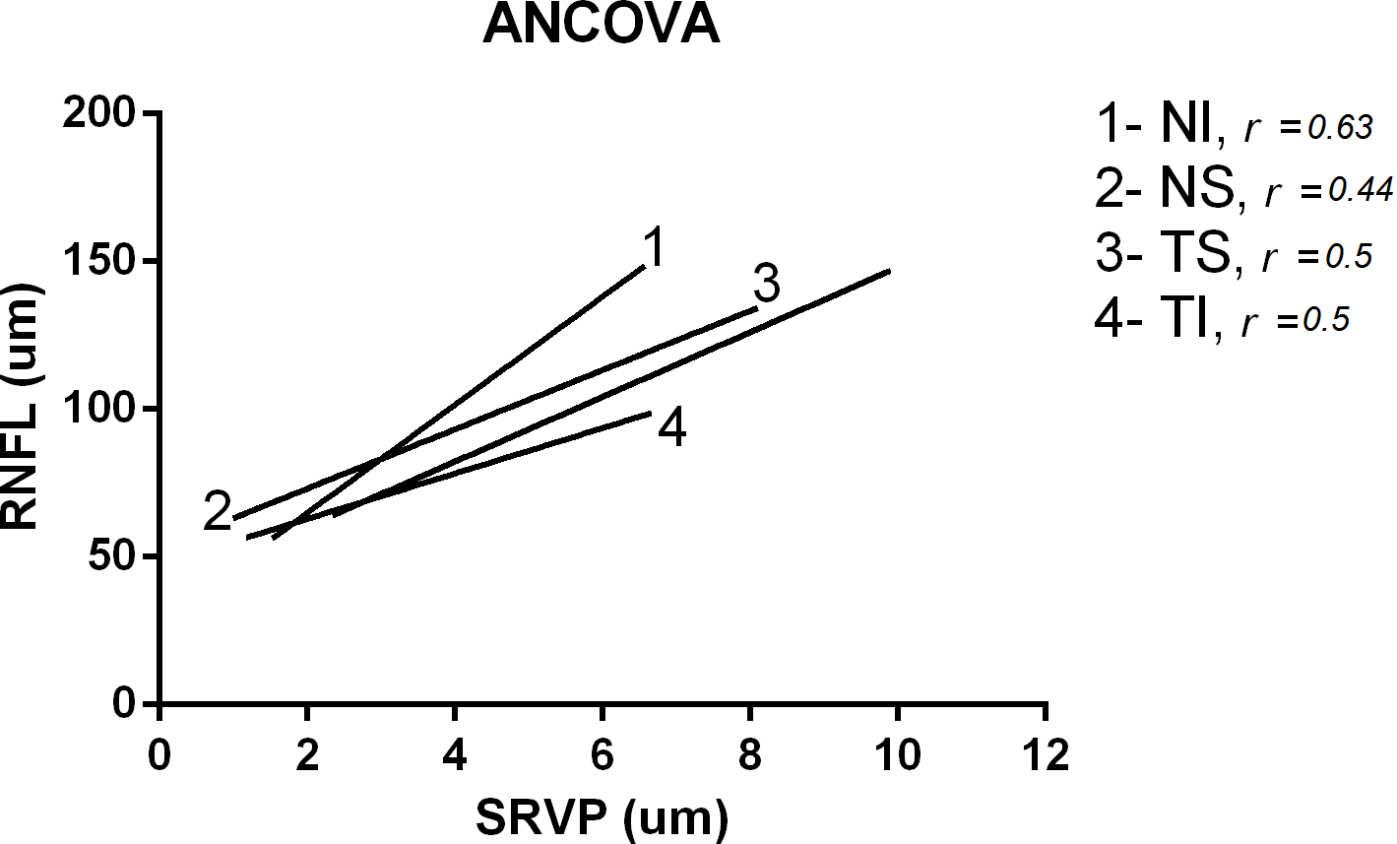
Results of the ANCOVA test applied for the four sectors when using linear regression. Correlation coefficient (r) is also shown.

Analysis of Covariance (ANCOVA) was used to study the significance of the linear regression slopes applied to the RNFL-SRVP relation in the four quadrants. Fig 4 shows the four linear regressions applied in the four sectors. Results of ANCOVA show that if the overall slopes were identical, there is a 25% chance of randomly choosing data points with slopes this different. We can conclude that the difference are not significant (p>0.05). Since the slopes are not significantly different, it is possible to calculate one slope for all data. The pooled slope equalled 10.8.

### Variability and venous calibre

**Fig 5:**
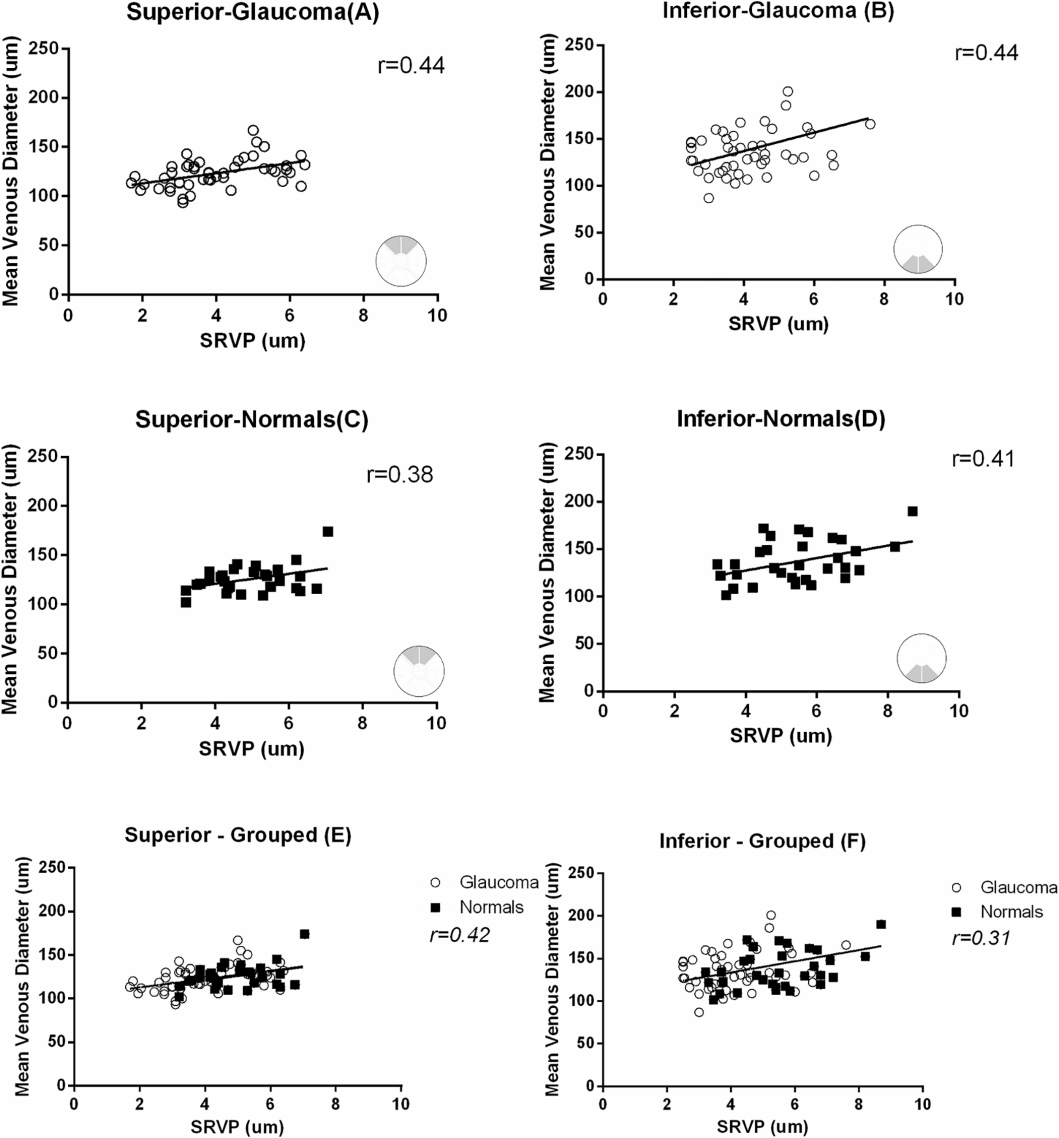
Correlation between average SRVP amplitude and mean venous diameter in the superior and inferior sectors both in glaucomatous and normal eyes. Grouped analysis for glaucoma subjects and normal controls in both sectors are also shown (E,F). Correlation coefficient for A,B,C, D,E and F are r = 0.44,0.44,0.36,0.41,0.42 and 0.31.

To assess any long-term fluctuation the mean superior and inferior SRVP amplitude and RNFL thickness for baseline and 6 month follow up are shown in Table 2. There was a non-significant change in RNFL and SRVP (p>0.05). We also observed a positive correlation between average SRVP amplitude and average venous diameter in the superior and inferior sectors both in glaucomatous and normal eyes. Fig 5 shows the results for this analysis. Correlations between RNFL thickness and SRVP amplitude, independent of venous diameter, were also examined by correcting SRVP amplitude values for venous diameter at 130 μm (i.e. average venous diameter). The corrected SRVP amplitude correlation with RNFL thickness showed minimal change, meaning the relationship is more dependent on RNFL.

## Discussion

In this study we investigated the relationship between nerve fibre layer thickness and retinal venous pulsatility. We investigated glaucoma subjects and normal controls. We found a significant difference (p<0.0001) between the two groups with lower SRVP amplitude in glaucoma. However, the relation also held in the controls despite a narrower range of RNFL thickness. (87±26 μm vs 111±15 μm, p<0.0001)

When analysis by sectors was performed, we observed lower SRVP amplitudes in those sectors with greater RNFL loss. It has been reported that ocular blood flow is reduced in glaucoma [18–20]. There are many potential reasons for this, and it may be either primary or secondary to reduced retinal demand.

Plange et al[21] showed a significant reduction in blood flow velocity of the central retinal vein (CRV) in glaucoma subjects. His findings also demonstrated a significant correlation between minimum velocities of CRV with RNFL cross sectional area (r^2^ = 0.43). A more recent study investigated the relationship between retinal blood flow and RNFL thickness[22]. An interesting finding of this study was that ocular blood flow was increased in regions of nerve fibre loss in the early stages of glaucoma and later reduced when the disease progressed. Reduced ocular blood flow in regions of RNFL loss in advanced glaucoma may also explain our findings which showed less SRVP in regions of thinner RNFL. However, the DVA cannot directly measure flow, so further studies would be required to confirm this.

Reduction of blood flow could be due to an increased venous resistance in glaucoma subjects [23]. A reduction in actual retinal vessel diameters may also contribute to an increase in vascular resistance [24] and we observed a positive correlation between SRVP amplitude, venous diameters and RNFL thickness. The Central Retinal Venous Equivalent (CRVE) and Central Retinal Arterial Equivalent (CRAE)[25], which are standardised measures of venular and arteriolar calibre, were 199±18 μm and 150±21 μm in our glaucoma subjects. These values were 203±17 and 156±25 μm in normals, respectively. While the difference between glaucoma and normal subjects is not significant (p = 0.5), our findings are consistent with previous population studies reporting narrower retinal venular and arteriolar diameter in glaucoma patients[26–28].

While the generation of SRVPs is not solely dependent on blood flow, reduced ocular blood flow and average venous diameter might contribute the finding of lower SRVP in regions of RNFL loss. We cannot determine from this study whether the reduction of SRVP is a primary event involved in the mechanism of glaucomatous damage (such as a raised central venous pressure), or secondary to reduced blood flow, or a combination of both factors.

Alteration of SRVP amplitude in glaucoma may also be influenced by other parameters such as thinning of lamina cribrosa and an increase in the trans-laminar pressure gradient. Jonas et al showed that the lamina cribrosa was significantly thinner in glaucoma patients compared with normals (p<0.001) and that the distance between intraocular space and the cerebrospinal fluid (CSF) space was significantly shorter (p<0.001)[29]. Previous studies have shown a greater trans-laminar pressure gradient in glaucoma subjects [14] and therefore one would expect an exposure of the intraocular space to the CSF space with greater pressure gradient will result in higher SRVP. However, this study and previous studies by Morgan et al [5] have shown that glaucoma subjects have less SRVP. Additionally Morgan et al[30] demonstrated that venous pulsation pressure, as measured by Ophthalmodynamometry, is significantly associated with risk of glaucoma progression. This finding was later confirmed by another study on independent cohort of 136 patients, which found a reduced SRVP in the progressing group (20% vs. 41%, p<0.05)[9].

Identification of SRVP clinically is a very subjective phenomenon and is influenced by microsaccades during observation, However this study is not reporting on visible SRVPs at the optic nerve head, but in the adjacent retinal veins beyond the neuroretinal rim. Our previous study established the waveform of these pulsations was identical to that recorded on the disc itself[8]. Clinical studies have reported a frequency of 75.3% [31] to 98%[5] in normals and 54%[5] to 64.1% in glaucoma patients. We observed an absence of visible SRVP in the majority of our glaucoma patients when screened by a 75D lens (74% of the patients were reported by the clinician to have no visible pulsations). However, when SRVP was recorded with the DVA, we observed measurable SRVP in all glaucoma patients including the most advanced cases. This demonstrates that although one may not be able to detect SRVP at the slit lamp, small amplitude SRVP still can be present, and monitoring it for change over time is feasible.

In this study we did not measure blood pressure and heart rate, however our previous studies have shown that blood pressure variations do not affect SRVP amplitude [8]. We are currently following these patients on a 6 monthly basis to further investigate the relation between SRVP and RNFL in progression.

We acknowledge that the distribution of nerve fibres originating in each of the optic disc sectors as defined by the OCT RNFL analysis does not exactly match the venous drainage distribution for the same vessel in that sector. We analysed various combinations of sectors and report here the best correlated sectors, which occurred when individual sectors were compared (eg TS RNFL correlated with the TS vein), or when both superior or both inferior veins were averaged and compared with the two corresponding RNFL sectors for superior or inferior RNFL.

Previous studies have shown acceptable reproducibility coefficients for the DVA, ranging from 1.3% to 5%, with reproducibility being slightly higher for retinal veins[32]. In our previous study, we tested the SRVP reproducibility in a placebo group and confirmed good reproducibility for SRVP amplitude measurements[8]. In the current study we tested the glaucoma subjects 6 months after their initial visit to determine if the relationship was consistent in subjects with glaucoma. The majority of subjects showed a non-significant decline in SRVP and RNFL over the 6 month follow up (p>0.05). It is possible the change could be due to fluctuation in IOP in the 6 month follow up tests, which is known to effect SRVP amplitude[8] or could represent alteration in blood flow or venous resistance associated with the process in glaucoma. Our results suggested a slight progression of the disease during the 6 month follow up in terms of loss of nerve fibre layer and an associated reduction in SRVP amplitudes. Long term studies will continue to define this relationship further.

While a lack of SRVP has been reported as a risk factor for glaucoma, other factors such as raised CSFp or lower IOP are also known to decrease SRVP amplitude. While we do not know the actual CSFp in our subjects, the IOP was normal in the controls, and in the glaucoma subjects IOP was under treatment and was in the normal range. We also do not think this is a drug effect directly on the vessel walls, since our previous study in normals of the acute effects of an alpha agonist and a beta blocker on SRVP did not show vascular effects[8]. Further longitudinal studies will reveal whether SRVP amplitude reduction progresses with the disease and the amplitude decreases with advancing nerve fibre loss, potentially making it a possible biomarker for progressive glaucoma.

In conclusion, SRVP amplitude shows a positive correlation with RNFL thickness in both glaucoma subjects and normal controls. is means that SRVP amplitude is reduced in areas where nerve fibre layer loss has occurred in glaucoma. This is more evident on the temporal veins where the nerve fibre loss is more dominant in glaucoma patients.
